# Analysis of the safety and efficacy of microwave ablation of several foci of multiple lung metastases from colorectal cancer

**DOI:** 10.3389/fonc.2025.1522470

**Published:** 2025-05-29

**Authors:** Bohan Song, Jinfeng Bai, Jinmei Zhou, Yinshan Yang, Qijie Wu, Ming Huang, Hongjie Fan, Xianshuo Cheng, Ping Liu, Yu-Dong Xiao, Xin Zhao, Chongying Deng, Shuai Luo, Rong Ding

**Affiliations:** ^1^ Department of Minimally invasive intervention, Yunnan Cancer Hospital, The Third Affiliated Hospital of Kunming Medical University, Kunming, China; ^2^ Department of Radiology, Union Hospital, Tongji Medical College, Huazhong University of Science and Technology, Wuhan, China; ^3^ Department of Colorectal Surgery, Yunnan Cancer Hospital, The Third Affiliated Hospital of Kunming Medical University, Kunming, China; ^4^ Department of Radiology, the Second Xiangya Hospital of Central South University, Changsha, China

**Keywords:** microwave ablation, colorectal cancer, lung metastasis, local treatment, efficacy and safety

## Abstract

**Objective:**

This study aims to retrospectively analyze the safety and effectiveness of microwave ablation (MWA) in treating multiple lung metastases from colorectal cancer. Additionally, it seeks to compare the superiority of single multiple ablation and fractionated multiple ablation for unilateral lung multiple metastases.

**Materials and methods:**

Retrospective analysis was conducted on clinical and pathological data of 82 patients with such multiple lung metastases from colorectal cancer treated from January 2020 to December 2022. Patients were categorized based on the number of MWA sessions required,Patients who had received only one MWA treatment were included in the single MWA group, and patients who had received two or more MWA treatments were included in the multiple MWA group. Chest-enhanced CT scans were performed at 1, 3, 6, and 12 months post-MWA to assess ablation outcomes. The primary focus was the median overall survival (mOS), while secondary endpoints encompassed median progression-free survival (mPFS), technical success rates, and safety. Analysis was performed by log-rank test and Cox proportional hazard regression model, using the Common Terminology Standard for Adverse Events (version 5.0) to assess safety within 28 days after MWA.

**Results:**

There were 82 patients with numerous lung metastases from colorectal cancer, and they had a total of 182 lesions. These patients underwent 112 microwave ablation (MWA) treatments. Each patient received at least two MWA treatments for their target lesions. The overall median overall survival (mOS) time for all patients was 25 months, the median progression-free survival (mPFS) time was 21 months. No deaths or severe adverse events occurred as a result of the treatment. The univariate Cox regression analysis indicated that fractional MWA (*P*=0.007) were adverse prognostic factors for CRC patients with lung metastasis.Upon accounting for various confounding factors, the significance of MWA times (*P*=0.006) remained pertinent in its association. Furthermore, the group that underwent single MWA showed a superior mOS compared to the group that underwent fractionated MWA (*P*=0.004).

**Conclusion:**

Microwave ablation proves to be a safe and efficacious treatment modality for colorectal cancer-associated multiple pulmonary metastases, offering substantial clinical benefits.

## Introduction

Colorectal cancer (CRC) ranks as the third most prevalent malignancy in terms of incidence but second in cancer-related mortality (1). The lungs represent the second most frequent site of CRC metastasis, preceded only by the liver. Surgical resection remains the standard curative approach for CRC-derived lung metastases (2). Evidence indicates that patients undergoing radical surgery for lung metastases achieve 5-year survival rates ranging from 35% to 70% (3–5). For those with unresectable pulmonary metastases, combination chemotherapy with targeted agents is the preferred therapeutic strategy. Notably, clinical studies have demonstrated enhanced efficacy with bevacizumab in combination with chemotherapy for CRC patients with lung metastases (6).

Currently, the management of unresectable CRC lung metastases typically involves a multimodal approach integrating systemic and local therapies. However, selected patients—particularly those with controlled primary lesions—may benefit from localized treatments to prolong survival. According to ESMO guidelines, stereotactic body radiotherapy (SBRT) and thermal ablation techniques are viable options for patients with unresectable CRC lung metastases (7). Reported outcomes indicate 3-year local control rates of 65–70.6% and 3-year survival rates of 56–64% in this patient population (8–10).

Radiofrequency ablation (RFA) and microwave ablation (MWA) are the most widely utilized thermal ablation modalities. Compared with RFA, MWA offers several theoretical and clinical advantages, including higher intratumoral temperatures, larger ablation zones, shorter procedure duration, and deeper tissue penetration (11). A meta-analysis of eight studies involving 230 patients demonstrated that MWA is an effective therapeutic option for colorectal cancer lung metastases and exhibits superiority over RFA in both theoretical properties and local tumor control rates (12). Notably, MWA has been associated with a median overall survival (OS) of 76 months in CRC patients with pulmonary metastases, with reported 1-, 2-, 3-, and 5-year survival rates of 93.5%, 80.6%, 61.3%, and 51.6%, respectively (13).

However, the management of bilateral multiple lung metastases remains a significant clinical challenge, with no established optimal treatment approach. Furthermore, limited data exist regarding the efficacy and safety of MWA for treating multifocal pulmonary metastases. Therefore, this study seeks to evaluate the therapeutic outcomes and safety profile of MWA in patients with multiple colorectal cancer lung metastases.

## Materials and methods

### Patients

We retrospectively analyzed the clinical and pathological data of 82 patients with multiple pulmonary malignancies who underwent microwave ablation (MWA) treatment at Yunnan Cancer Hospital and Union Hospital, Tongji Medical College, Huazhong University of Science and Technology between January 2020 and December 2022. Prior to MWA intervention, all cases were evaluated by our multidisciplinary team (MDT) to confirm diagnosis and assess target lesions.

The treatment strategy was individualized based on patient characteristics. Patients eligible for single-session complete ablation received one MWA procedure targeting all pulmonary lesions. For cases with large tumor burden, compromised pulmonary function, technical challenges in achieving complete ablation, or evidence of intrapulmonary progression post-ablation, staged MWA procedures were performed. In bilateral lung involvement, the decision between single-session or staged treatment was made considering both clinical factors and patient preference. When bilateral single-session ablation was not feasible, we implemented a sequential approach with separate MWA procedures for each lung.Written informed consent was obtained from all participants prior to treatment.

### Selection of patients

Each patient’s preoperative assessment encompasses a chest-enhanced CT scan, abdominal ultrasound scan, electrocardiogram, echocardiogram, and pulmonary function tests. Fluorine 18 fluorodeoxyglucose (FDG) PET scans were conducted in select cases. The patient inclusion criteria are as follows: (1) Age ≥ 20 years, with an Eastern Cooperative Oncology Group (ECOG) performance status of 0 or 1. (2) The primary lesion was resected without local recurrence. (3) The nodules are consistent with lung metastases and have not invaded other organs. (4) The number of lesions in the lungs is equal to or greater than 2 and less than 5. (5) Lung metastases are less than 5cm in diameter. (6) Feasibility of performing MWA with technical success. (7) Absence of abnormal laboratory results, including platelet count > 100,000/ml, prothrombin time < 18 seconds, and prothrombin activity > 40%. Patients failing to meet these criteria will be excluded from the analysis, including those with malignant lung tumors that do not fulfill the specified requirements.

The preoperative objective was to achieve complete ablation of all target lesions in a single procedure, with such cases classified as the single ablation group. Patients who demonstrated intraoperative intolerance, had lesions too extensive for complete ablation, or met other exclusion criteria for single-session treatment were offered staged ablation after thorough discussion, and these cases were allocated to the multiple ablation group.

### MWA for the lung

#### Preoperative patient preparation

Patients are required to abstain from consuming solid food for a period of 8 hours prior to procedure, and from consuming liquid food within 4 hours preceding the procedure. Prior to surgery, venous access is established as part of the preoperative preparation. Surgical skin preparation may be performed if deemed necessary. Following routine disinfection, local anesthesia using 1% lidocaine hydrochloride is typically administered. Preoperative sedation and analgesia are achieved with a combination of 2ml diazepam and 2ml sufentanil citrate. In case of patient discomfort during the operation, intravenous administration of sufentanil citrate (0.5-1 μg/kg/min) is continued.

#### Procedural

Microwave ablation (MWA) of the lung was conducted under CT guidance, with ablation performed subsequent to the identification of target lesion location through CT scans. This procedure was carried out by six interventional radiologists, each possessing over a decade of experience. Utilizing the KY-2200 microwave ablation therapy device (Canyon, Nanjing, China), operating at a frequency of 2450MHz ± 50MHz and with an output power range of 0 to 100W. The ablation needles employed were KY-2450B-T1 disposable microwave ablation needles, characterized by a diameter of 1.8 ± 0.3 mm, a shaft length of 10 ± 3 cm, and an active tip of 12 ± 3 mm.

The ablative power was adjusted within a range of 30-60W based on tumor size, with individual tumor ablation time maintained between 2-10 minutes. Following each ablation session, a CT scan was performed to determine whether adjustments to the needle angle, continued power application, or cessation of ablation were necessary before withdrawing the needle. Due to medical-related cost reasons, the same ablation needle is used to treat all target lesions during each MWA treatment. After each lesion is ablated, the needle is completely disinfected before treating the next lesion. For adjacent lesions, the needle tip can be adjusted for ablation, while for lesions that are further apart, the ablation needle needs to be removed and disinfected before another ablation can be performed. During needle removal, the needle tip maintained its original power level to prevent potential implantation metastasis of tumor cells. The success of the technique was defined by the completion of the treatment plan, corroborated by follow-up CT scans one month post-MWA, demonstrating comprehensive coverage of the tumor by the ablation area.

In cases of pleural complications, asymptomatic patients with minimal fluid accumulation (unilateral effusion <200 mL) may be managed conservatively with close observation. However, immediate closed thoracic drainage is indicated if patients exhibit significant symptoms (e.g., dyspnea or hypoxemia) or if imaging reveals a unilateral effusion occupying ≥20% of the hemithorax. For pleural effusions ≥500 mL (or radiologically confirmed ≥1/3 unilateral chest volume) accompanied by dyspnea, therapeutic drainage is required. In patients with hemoptysis, minor bleeding may be controlled via puncture tract embolization using gelatin sponge particles. However, progressive hemorrhage (>200 mL/h) or massive bleeding (>500 mL) necessitates urgent surgical intervention (14).

In patients presenting with post-MWA fever (body temperature >38°C), immediate evaluation should include continuous temperature monitoring, laboratory tests (complete blood count, sputum/blood cultures), and contrast-enhanced CT imaging. Empirical antibiotic therapy should be initiated and subsequently adjusted based on culture and sensitivity results. The recommended treatment duration is typically 2–4 weeks, with extended courses required for complicated infections. For cases complicated by empyema, CT-guided drainage is indicated. Follow-up CT imaging at 4–6 weeks post-treatment is essential to assess lesion resolution.

#### Follow-up

Chest enhancement CT scans were performed at 1, 3, 6, and 12 months after the completion of MWA to evaluate the ablation effect. If necessary, PET-CT can be performed for a more accurate assessment of treatment efficacy and disease progression. The follow-up ended on March 1, 2023. Prognostic factors taken into consideration encompassed age, gender, BMI (Body Mass Index), maximum tumor diameter, number of pulmonary metastases, primary tumor location, target lesion location, levels of CEA, CA199, and CA125 during the initial MWA, primary tumor’s T stage, number of underlying health conditions, internal lung tumor environment, and presence of extrapulmonary metastasis.

During follow-up, patients continued to receive the initial treatment regimen. Disease progression prompted a transition to second-line therapy in accordance with evidence-based guidelines for colorectal cancer management.

#### End point

The primary endpoint focused on determining the median overall survival (OS) time, where OS time was defined as the duration between the initial lung microwave ablation (MWA) and either the date of death from any cause or the last follow-up. Secondary endpoints included examining prognostic factors, technical success rates, progression-free survival (PFS), and safety measures. The chemotherapy, targeted therapy, and 125I particle implantation treatment was allowed. Tumors that are not completely or partially progressed but meet the selection criteria will receive MWA treatment again after at least 28 days of ablation therapy. To evaluate safety within the 28 days post-MWA, use the Common Terminology Criteria for Adverse Events (CTCAE - 5.0 version) (8) to assess the severity of treatment-related adverse events.

#### Statistical analysis

In this study, quantitative data were presented as either the mean ± standard deviation or the median. To compare two groups, either an independent Student’s t-test or the Mann-Whitney U test was employed, depending on the distribution of the data. Cumulative survival and local tumor progression curves were generated using the Kaplan-Meier method, and these curves were juxtaposed through the log-rank test. In this study, prognostic factors were investigated using both univariate and multivariate Cox proportional hazards regression models. The differences between the two groups were reported as hazard ratios (HRs) and were accompanied by 95% confidence intervals (CIs). All reported P values were two-sided, where values below 0.05 were deemed significant. Statistical analysis, baseline data comparisons, and Cox regression analysis were performed using SPSS (version 26.0; IBM Corp).

## Results

### Participants

Between January 2020 and December 2022, 82 patients with CRC patients with multiple pulmonary metastasis were enrolled in this study (**Table 1**), treat at least two tumors each time. Forty patients received a single MWA, while 42 patients underwent staged MWA, and 182 MWA treatments were performed. A summary of participant and tumor characteristics is given in **Table 1**. The age range is 30-85 years old, with a median age of 58 years old. These included 59 male patients (71.95%) and 23 female patients (28.05%). In this study, 13 patients had 3 lung metastases (15.85%), 63 patients had 3-5 lung lesions (76.83%), and 6 patients had more than 5 lung lesions (7.31%). The median follow-up time was 20 months (95% CI:14.27–23.73).

**Table 1:** Demographic and clinical characteristics of the study population.

Characteristics		Ablation times		
	Total, n=82	Single, n=40, 48.8%	Multiple, n=42, 51.2%	P-value
Age, Median ± SD	59 ± 11.96	59 ± 12.02	57 ± 9.54	
Age, n (%)				0.117
≤65	61	32 (80)	23 (55)	
>65	21	8 (20)	13 (45)	
Gender,n (%)				0.381
Female	23	13 (32.5)	10 (23.8)	
Male	59	27 (67.5)	32 (76.2)	
BMI				
≤24	81	40 (100)	41 (97.7)	
>24	1	0 (0)	1 (2.3)	
T stage,n (%)				0.136
T1-T2	27	10 (25)	17 (40.5)	
T3-T4	55	30 (75)	25 (59.5)	
Extrapulmonary metastasis				0.812
Yes	30	18 (45)	20 (47.6)	
No	52	22 (55)	22 (52.4)	
Location,n (%)				0.524
Center	6	1 (2.5)	5 (11.9)	
Periphery	53	26 (65)	27 (64.3)	
Diffusion	23	13 (32.5)	10 (23.8)	
Number of Lesions,n (%)				0.265
<3	13	4 (10)	9 (21.4)	
3-5	63	35 (87.5)	28 (66.7)	
>5	6	1 (2.5)	5 (11.9)	
Maximum tumor diameter,n (%)				0.685
>3cm	5	2	3	
≤3cm	77	38	39	
Nature of lesion,n (%)				0.256
GGO	9	6 (15)	3 (7)	
Solid	59	30 (75)	29 (69)	
Mix	14	4 (10)	10 (23.8)	
CEA,n (%)				0.837
Negative	44	21 (52.5)	23 (54.8)	
Positive	38	19 (47.5)	19 (45.2)	
CA125,n (%)				0.951
Negative	76	37 (92.5)	39 (92.9)	
Positive	6	3 (7.5)	3 (7.1)	
CA199,n (%)				0.108
Negative	69	31 (77.5)	38 (90.5)	
Positive	13	9 (22.5)	4 (9.5)	

BMI, Body Mass Index; GGO, ground glass opacity; CEA, carcinoembryonic antigen; CA125, carbohydrate antigen 125; CA199, carbohydrate antigen199.

### Survival

**Figure 1:**
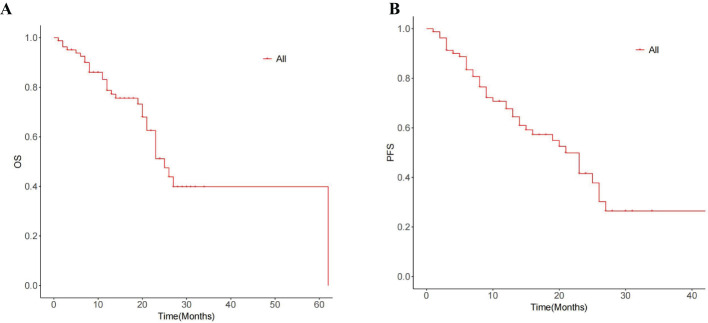
Graph shows the median OS time **(A)** and median PFS time **(B)** for the entire cohort.

**Figure 2:**
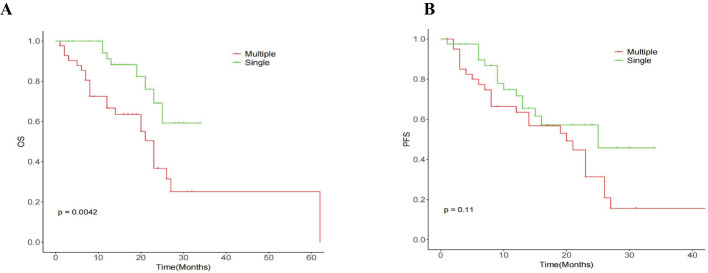
Graph shows the median OS time **(A)** and median PFS time **(B)** for the entire cohort.

The median OS for the whole group is 25 months(95% CI:20.88, 29.12) (**Figure 1A**). The median overall survival (OS) in the single-session MWA group did not surpass 23 months. In the multiple-session MWA group, the median OS stood at 23 months (95% CI:19.95,26.05), revealing a statistically significant difference (P=0.004; **Figure 2A**). The univariate Cox regression analysis demonstrated that sequential MWA (P<0.05) were risk factors for death in patients with Colorectal cancer(CRC). After adjusting for different confounding factors, sequential MWA(HR,5.279; 95% CI:0.112,11.692;P =0.006) was considered a risk factor for death (**Table 2**).

**Table 2:** Relationship between the overall survival of a single MWA and multiple MWA in CRC patients.

			Multivariate analysis						
Variables	Univariate analysis		Model A		Model B		Model C		
	HR (95% CI)	*p*	HR (95% CI)	*p*	HR (95% CI)	*p*	HR (95% CI)		*p*
Gender									
Female	1[Reference]		1[Reference]		1[Reference]		1[Reference]		
Male	0.247 (0.240,1.443)	0.731	0.691 (0.277,1.721)	0.427	0.647 (0.23,1.814)	0.408	0.484 (0.154,1.521)		0.214
Age									
≤65	1[Reference]		1[Reference]		1[Reference]		1[Reference]		
>65	0.845 (0.562,1.271)	0.419	0.705 (0.309,1.606)	0.405	0.555 (0.197,1.558)	0.264	0.701 (0.241,2.033)		0.513
BMI									
≤24	1[Reference]		1[Reference]		1[Reference]		1[Reference]		
>24	21.32 (0.001,475.19)	0.549	5.695 (0.522,15.689)	0.981	1.254 (0.054,9.512)		12.52 (2.365,22.322)		0.981
T stage									
T1-T2	1[Reference]				1[Reference]		1[Reference]		
T3-T4	1.466 (0.708,3.034)	0.303			1.357 (0.568,3.243)	0.492	1.436 (0.585,3.523)		0.429
Extrapulmonary metastasis									
No	1[Reference]				1[Reference]		1[Reference]		
Yes	1.81 (0.804,4.076)	0.152			1.858 (0.738,4.674)	0.188	2.364 (0.851,6.566)		0.099
Comorbidity,n (%)									
No	1[Reference]				1[Reference]		1[Reference]		
Yes	0.611 (0.297,1.258)	0.181			0.930 (0.220,3.934)	0.921	0.519 (0.075,3.614)		0.753
Location									
Center	1[Reference]				1[Reference]		1[Reference]		
Periphery	0.505 (0.107,2.376)	0.387			0.105 (0.018,0.618)	0.313	13.461 (0.148,60.046)		0.221
Diffusion	0.548 (0.096,3.136)	0.499			0.751 (0.261,2.159)	0.595	0.646 (0.221,1.886)		0.424
Maximum tumor diameter,n (%)									
≤3cm	1[Reference]				1[Reference]		1[Reference]		
>3cm	2.424 (0.329,17.858)	0.385			0.639 (0.054,5.405)	0.6	0.477 (0.046,4.928)		0.535
Number of Lesions,n (%)									
<3	1[Reference]				1[Reference]		1[Reference]		
3-5	1.016 (0.239,4.33)	0.982			1.737 (0.193,15.602)	0.622	0.705 (0.125,3.980)		0.822
>5	0.858 (0.166,4.438)	0.855			0.863 (0.077,9.649)	0.904	3.217 (1.139,9.085)		0.627
Nature of lesion									
GGO	1[Reference]				1[Reference]		1[Reference]		
Solid	1.095 (0.322,3.724)	0.884			0.764 (0.040,14.650)	0.858	1.301 (0.131,12.899)	0.692	
Mix	2.337 (1.049,5.806)	0.838			0.298 (0.037,2.412)	0.256	0.605 (0.048,7.549)	0.075	
Pneumonectomy									
No	1[Reference]				1[Reference]		1[Reference]		
Yes	2.216 (0.670,7.33)	0.192			2.277 (0.595,8.711)	0.229	2.005 (0.516,7.7955)	0.315	
CEA									
Positive	1[Reference]						1[Reference]		
Negative	0.848 (0.411,1.75)	0.655					1.684 (0.603,4.697)	0.32	
CA125									
Positive	1[Reference]						1[Reference]		
Negative	2.1 (0.731,6.035)	0.168					1.26 (0.276,5.742)	0.765	
CA199									
Positive	1[Reference]						1[Reference]		
Negative	1.022 (0.355,2.94)	0.968					1.889 (0.457,7.809)	0.38	
Ablation times									
Single	1[Reference]		1[Reference]		1[Reference]		1[Reference]		
Multiple	1.739 (1.159,2.608)	0.007	1.32 (0.142,2.723)	0.006	4.2810.116,38.254)	0.005	5.279 (0.112,11.692)	0.006	

HR, hazard ratio; CI, confidence interval; CEA, carcinoembryonic antigen; CA125, carbohydrate antigen 125; CA199, Carbohydrate antigen199.

Kaplan-Meier survival analysis shows that the median PFS for the whole group is 21 months (95% CI:15.21,26.79) (**Figure 1B**). The single MWA group did not reach the median PFS time; the median PFS of the multiple group was 25 months (95% CI:10.02, 29.98;P=0.111; **Figure 2B**).

### Safety

**Figure 3:**
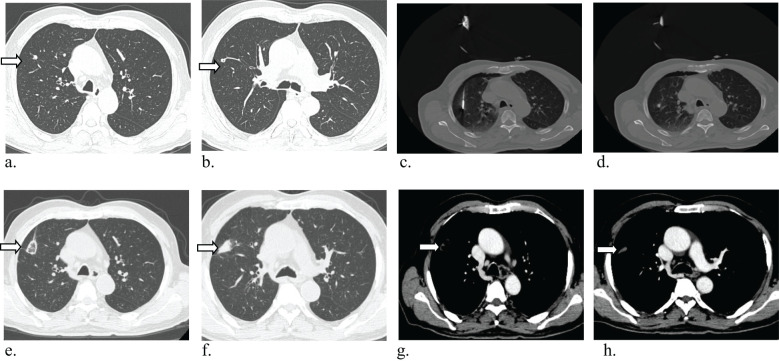
Axial CT images in 81-year-old man with multiple lung metastases from colorectal cancer, and the CRC resected 5 months ago. **(a, b)** Baseline image shows Baseline imaging shows two solid lung nodules in the right upper lobes(arrow), radiographic examination indicated lung metastases. **(c, d)** Microwave ablation (MWA) treatment for two lesions. **(e–h)** CT image obtained one month after MWA shows that the tumor was well covered by ablative zone (arrow).

The procedure achieved technical success in all instances (**Figure 3**), with no surgical-related fatalities or grade 3 or 4 adverse events as per CTCAE 4.03. The most prevalent complication observed was pneumothorax, which manifested in 13 cases; among them were 7 cases in the single MWA group and 6 cases in the multiple MWA group. Among these cases, 5 patients necessitated closed drainage, two in the single group and 2 in the multiple group. Furthermore, six patients encountered postoperative pain, and five experienced a minor pleural effusion, two requiring closed drainage. Additionally, four patients developed postoperative fever, and two exhibited a minor hemoptysis. There is no significant difference in the occurrence of complications between the two groups (P>0.05, **Table 3**).

**Table 3:** Complication between two groups.

Group	Pneumothorax	Pleural effusion	pain	Infect	Hemoptysis	Total
Single	7	3	4	3	1	17
multiple	6	2	2	1	1	12
χ^2^-value	0.21	0.10	0.53	0.53	0.433	0.43
P-value	0.65	0.75	0.47	0.47	1.00	0.21

## Discussion

While surgical resection continues to be a viable treatment option for pulmonary metastases in colorectal cancer, its clinical application faces several limitations. The efficacy of surgical intervention becomes particularly controversial in cases involving: (1) more than three metastatic lesions, (2) bilateral pulmonary involvement, (3) deeply located metastases, (4) significant patient comorbidities, (5) concurrent distant metastases at other sites, (6) short disease-free intervals between primary tumor resection and metastatic progression, or (7) pulmonary metastases detected during systemic chemotherapy (15–17).

Over the past decade, image-guided therapies have gained increasing recognition in oncology. Among these, thermal ablation techniques—including MWA, RFA, and laser-induced thermal therapy (LITT)—have emerged as established therapeutic options for unresectable primary and metastatic lung malignancies (18–21). Current evidence supports the safety and efficacy of percutaneous ablation modalities, particularly for patients ineligible for surgical resection due to comorbidities, advanced disease, or compromised pulmonary function.

Recent comparative studies have evaluated the outcomes of LITT and MWA in patients with colorectal cancer lung metastases, with a focus on local tumor control, time to progression, and overall survival rates. Statistical analyses suggest that MWA may offer superior local tumor control rates compared to LITT, highlighting its potential clinical advantages for this patient population (17). Nagore et al. (22) demonstrated no statistically significant difference in OS or local tumor control between stereotactic body radiotherapy (SBRT) and surgical resection for colorectal cancer lung metastases, suggesting comparable therapeutic efficacy for these two treatment modalities in this patient population,and overall survival was significantly different between patients with multiprogression and those with oligoprogression (median survival of 24.48 and 71.23 months, P < 0.001, 95% CI 50.35–92.11 months).

Thermal ablation technology has become prevalent in the treatment of colorectal cancer (CRC) lung metastases, with numerous studies investigating its safety and efficacy. However, its efficacy remains controversial.Many studies have shown that for patients with oligometastatic disease, radical local treatment can bring survival benefits (15).Research by Takaaki Hasegawa demonstrated that for resectable CRC lung metastases with diameters of 3 cm or less, the 3-year overall survival rate following lung RFA reached 84% (23). Nevertheless, to our knowledge, there have been no studies reporting the safety and effectiveness of MWA in treating multiple CRC lung metastases.

Due to its minimally invasive and repeatable advantages, MWA is frequently chosen for treating multiple, recurrent, or new metastatic diseases. An example is the study by Guanghui Huang et al., which documented the treatment of 33 patients with 103 ground-glass opacity (GGO) lesions using microwave ablation (MWA). All patients remained alive with no signs of local progression or tumor recurrence by the end of the follow-up period. This study suggests that CT-guided percutaneous MWA for multiple synchronous lung GGO lesions is both feasible and effective in the short term (24). It has been reported that the median survival time of 32 patients with lung metastases from colorectal cancer treated with MWA was 31 months (25). The size and number of lung metastases in colorectal cancer have been identified as prognostic factors for patient survival (26). A study of 293 colorectal cancer patients with lung metastases revealed that lung metastases greater than 2 cm in size (HR = 2.10, P = 0.0027) and a number of metastases of 3 or more (HR = 1.86, P = 0.011) were significantly associated with local control and overall survival (27). Our research findings indicate a median overall survival (OS) of 26 months and a median progression-free survival (PFS) of 23 months in the treatment of multiple malignant lung tumors using MWA, which is shorter than previously reported (13, 22, 25), there remains room for further improvement.

The outcomes of this study indicate that among patients with multiple malignant lung tumors, single microwave ablation (MWA) surpasses multiple MWA in overall survival (OS), with a statistically significant difference. However, there is no significant difference in progression-free survival (PFS) and complication rates between the two groups. Several factors contribute to this phenomenon. Multiple MWA treatments often necessitate numerous punctures, increasing the risk of puncture-related complications such as bleeding, pneumothorax, and infection. Conversely, a single MWA procedure requires only one puncture, thereby mitigating the risk of complications (28–30).Single MWA provides superior protection to surrounding tissues and organs. In contrast, multiple MWA treatments may cause tissue trauma and damage. Earlier studies have shown that for larger tumors, increased punctures and ablations lead to more significant lung tissue damage, possibly resulting in a higher incidence of pneumonia (28). Single MWA allows for more controlled and minimized damage to adjacent structures. Additionally, while tumors with diameters greater than 3 cm may require multiple MWA sessions for eradication, a single MWA treatment can effectively eliminate tumor cells in one intervention, reducing the risk of tumor recurrence and metastasis, and ultimately enhancing patient survival rates (31). Finally, multiple MWA treatments could potentially induce the expression of tumor-related factors, fostering tumor recurrence or metastasis (32). MWA of lung tumors may stimulate distant extrapulmonary subcutaneous tumor growth, promote tumor proliferation and angiogenesis, which results in shorter survival times for patients with lung tumors.In this study, patients with multiple lung target lesions within the same lung segment were treated with a single puncture and multi-point ablation. Target lesions near the interlobar fissure were punctured parallel to the interlobar fissure. The puncture path was carefully chosen to maximize distance from the pericardium and major cardiac vasculature. If the lesion location presents a high surgical risk, coaxial puncture needles are recommended for ablation. If pulmonary hemorrhage occurs, removal of the ablation needle is not advised. The ablation needle can be extended and injected with a gelatin sponge embolic agent or other hemostatic drugs to control bleeding, and the needle can be removed once symptoms stabilize. If symptoms worsen, immediate embolization of the lower bronchial artery with DSA is recommended.

It is important to acknowledge certain limitations of this study. As a retrospective single-institution investigation with a limited sample size, this study is subject to inherent constraints. Comprehensive pulmonary function tests were not conducted for all patients undergoing MWA, and the follow-up period remains relatively brief. Consequently, forthcoming prospective, multicenter, randomized controlled trials are essential to ascertain the safety and efficacy of MWA in treating multiple lung metastases from CRC. Additionally, these trials could further investigate the optimal number of MWA treatment courses.

## Conclusion

Our clinical experience demonstrates that microwave ablation (MWA) represents a safe and effective therapeutic approach for managing multiple pulmonary metastases in colorectal cancer patients. The procedure is associated with favorable clinical outcomes, including an acceptable safety profile with low complication rates and potential survival benefits, highlighting its significant clinical utility in this patient population. While preliminary data suggest a potential survival advantage with single-session MWA over multi-session approaches, treatment selection should be individualized, incorporating patient-specific tumor characteristics, functional reserve, and multidisciplinary clinical evaluation to optimize outcomes.

## Data Availability

Publicly available datasets were analyzed in this study. This data can be found here: https://doi.org/10.6084/m9.figshare.24309805.v1.
